# Application of a remote-sensing three-source energy balance model to improve evapotranspiration partitioning in vineyards

**DOI:** 10.1007/s00271-022-00787-x

**Published:** 2022-04-05

**Authors:** Vicente Burchard-Levine, Héctor Nieto, William P. Kustas, Feng Gao, Joseph G. Alfieri, John H. Prueger, Lawrence E. Hipps, Nicolas Bambach-Ortiz, Andrew J. McElrone, Sebastian J. Castro, Maria Mar Alsina, Lynn G. McKee, Einara Zahn, Elie Bou-Zeid, Nick Dokoozlian

**Affiliations:** 1 Environmental Remote Sensing and Spectroscopy Laboratory (SpecLab), Spanish National Research Council (CSIC), Madrid, Spain; 2 Institute of Agricultural Sciences, Spanish National Research Council (CSIC), 28006 Madrid, Spain; 3 Agriculture Research Service, U.S. Department of Agriculture, Hydrology and Remote Sensing Laboratory, Beltsville, MD 20705 USA; 4 USDA-ARS, National Laboratory for Agriculture and the Environment, Ames, IA USA; 5 Department of Plants Soils and Climate, Utah State University, Logan, UT USA; 6 Department of Land, Air and Water Resources, University of California, Davis, CA USA; 7 USDA-ARS Crops Pathology and Genetics Research Unit, Davis, CA USA; 8 Department of Viticulture and Enology, University of California, Davis, CA USA; 9 E and J Gallo Winery, Winegrowing Research, Modesto, CA USA; 10 Department of Civil and Environmental Engineering, Princeton University, Princeton, NJ USA

## Abstract

Improved accuracy of evapotranspiration (ET) estimation, including its partitioning between transpiration (T) and surface evaporation (E), is key to monitor agricultural water use in vineyards, especially to enhance water use efficiency in semi-arid regions such as California, USA. Remote-sensing methods have shown great utility in retrieving ET from surface energy balance models based on thermal infrared data. Notably, the two-source energy balance (TSEB) has been widely and robustly applied in numerous landscapes, including vineyards. However, vineyards add an additional complexity where the landscape is essentially made up of two distinct zones: the grapevine and the interrow, which is often seasonally covered by an herbaceous cover crop. Therefore, it becomes more complex to disentangle the various contributions of the different vegetation elements to total ET, especially through TSEB, which assumes a single vegetation source over a soil layer. As such, a remote-sensing-based three-source energy balance (3SEB) model, which essentially adds a vegetation source to TSEB, was applied in an experimental vineyard located in California’s Central Valley to investigate whether it improves the depiction of the grapevine-interrow system. The model was applied in four different blocks in 2019 and 2020, where each block had an eddy-covariance (EC) tower collecting continuous flux, radiometric, and meteorological measurements. 3SEB’s latent and sensible heat flux retrievals were accurate with an overall RMSD ~ 50 W/m^2^ compared to EC measurements. 3SEB improved upon TSEB simulations, with the largest differences being concentrated in the spring season, when there is greater mixing between grapevine foliage and the cover crop. Additionally, 3SEB’s modeled ET partitioning (T/ET) compared well against an EC T/ET retrieval method, being only slightly underestimated. Overall, these promising results indicate 3SEB can be of great utility to vineyard irrigation management, especially to improve T/ET estimations and to quantify the contribution of the cover crop to ET. Improved knowledge of T/ET can enhance grapevine water stress detection to support irrigation and water resource management.

**Supplementary Information:**

The online version contains supplementary material available at 10.1007/s00271-022-00787-x.

## Introduction

As in many arid and semi-arid regions, agricultural production in California is facing the important challenge of meeting production goals while managing greater competition and uncertainty of the limited water resources available, which are affected by greater inter-annual variability of rainfall (Kustas et al. 2018; Wilson et al. 2016). Despite long-term droughts exacerbating water stress and competition, grape production is still strong in California, representing close to 350,000 ha and valued at around 5 billion USD (California Department of Food and Agriculture and USDA National Agricultural Statistics Service 2020). As such, there is an increasing interest and need to improve water resources management, notably in developing efficient irrigation management strategies that can be applied to vineyards.

Evapotranspiration (ET), the sum of the soil/surface evaporation (E) and plant transpiration (T), is a highly important process to understand, monitor, and detect water stress in crops. Thermal remote sensing, coupled with surface energy balance (SEB) models, has been demonstrated as robust methods to assess ET at different spatial and temporal scales (Anderson et al. 2020; Gerhards et al. 2019; Kustas and Anderson 2009). They offer spatially explicit information that can provide valuable information to support irrigation strategies in vineyards (Bellvert et al. 2020). SEB models compute ET, or the latent heat flux (LE) in energy terms, as the residual of the energy balance. The available energy, net radiation (Rn) minus ground heat flux (G), is partitioned into the sensible heat flux (H) and LE. Among different SEB models, the two-source energy balance (TSEB; Norman et al. 1995) model has been robustly applied in vineyards (e.g., Knipper et al. 2019; Nieto et al. 2019a, b) and other landscapes (e.g., Andreu et al. 2018; Li et al. 2019). Compared to one-source SEB models, TSEB additionally explicitly partitions LE between the vegetation (i.e., T) and soil (i.e., E) sources. This is especially relevant within an agronomic point of view, where irrigation strategies may want to reduce water losses from E compared to T, since vegetation T is intrinsically linked to crop biomass production. However, despite growing research interest in ET partitioning (T/ET; Nelson et al. 2020; Stoy et al. 2019), separating T and E remains a challenge due to their similar signals and complex relation with soil moisture, meteorology, and plant physiology (Perez-Priego et al. 2018; Scott and Biederman 2017). Several *in situ* T/ET measurements techniques have been developed (Anderson et al. 2017; Kool et al. 2014); however, these often rely on tenuous assumptions and are not spatially distributed nor inferable to the larger landscape or regional scales.

The 3SEB model, developed in Burchard-Levine et al. (2022), added a vegetation source (overstory vegetation + understory vegetation + soil) to the TSEB model. 3SEB was originally designed for tree-grass agro-forests or savanna ecosystems, which have distinct overstory and understory vegetation. However, vineyard landscapes are also characterized by clumped tall vine foliage along with a wide interrow system that may have the presence of herbaceous cover crops. As such, the 3SEB model structure may also serve to improve the modeling representation of vineyards compared to TSEB’s dual-source representation. Three-source energy-combination models have been developed to compute ET in vineyards, notably to directly account for both grapevine and interrow effects on the energy balance (Montes et al. 2014; Poblete-Echeverría and Ortega-Farias 2009). However, a more simplified thermal remote-sensing-based model, such as 3SEB, has not yet been assessed in these landscapes. Therefore, the main objective of this study is to apply 3SEB over a vineyard study site in California, USA, to investigate and quantify the ET partitioning between the different landscape sources, including the vine foliage and interrow cover crop. 3SEB results were also compared against TSEB simulations. This work was done under the framework of the Grape Remote-sensing Atmospheric Profile and Evapotranspiration eXperiment (GRAPEX) project, which aims to develop remote-sensing techniques to monitor ET from the sub-vineyard to regional scales (Kustas et al., 2018). TSEB and 3SEB were applied at the local tower footprint level over an experimental vineyard site in the Madera county of California´s Central Valley using data collected in 2019 and 2020. The main goal of the present study is to provide insights into the utility of 3SEB to improve vineyard stress detection, allowing to manage irrigation and water use more efficiently in these complex and valuable landscapes.

## Materials and methods

### Three-source energy balance (3SEB) model

Burchard-Levine et al. (2022) developed a remote-sensing-based three-source energy balance model (3SEB), which adds a vegetation source to the widely used two-source energy balance (TSEB) model (Kustas and Norman 1999a, b; Norman et al. 1995). 3SEB was developed to account for the multiple and contrasting vegetation layers in heterogeneous landscapes, such as tree-grass or savanna ecosystems. The vineyard (vine + interrow cover crop) system also has complex features in which the 3SEB may enhance its representation compared to the traditional two-source representation.

Assuming blackbody emissivity, radiometric land surface temperature (T_rad_) is separated into three components: soil temperature (T_soil_), understory (i.e., cover crop) temperature (T_un_), and overstory (i.e., vine) temperature (T_ov_) (Eq. 1)

1.1 $$T_{rad} = \left[ {f_{\left( \theta \right),ov} T_{ov}^{4} + \left( {1 - f_{\left( \theta \right),ov} } \right)T_{sub}^{4} } \right]^{1/4}$$

1.2 $$T_{sub} = \left[ {f_{\left( \theta \right),un} T_{un}^{4} + \left( {1 - f_{\left( \theta \right),un} } \right)T_{soil}^{4} } \right]^{1/4} \,,$$

where T_sub_ is the substrate (understory vegetation + soil) temperature; $f_{\left( \theta \right), c}$ is the fraction of vegetation (*c* for either understory, *un*, or overstory, *ov*) observed by the sensor and estimated as: $f_{\left( \theta \right),c} = 1 - exp\left( { - k_{bc} \Omega_{c} F_{c} } \right)$, where F is the local leaf area index (LAI; m^2^/m^2^), $\Omega$ is the clumping index (−), and $k_{b}$ is the beam extinction coefficient described in Campbell and Norman (1998). Using this procedure, the energy balance is thus solved over the three main landscape sources: overstory vegetation (i.e., vine foliage), understory vegetation (i.e., cover crop), and soil (Eq. 2)

2.1 $$LE_{ov} = Rn_{ov} - H_{ov}$$

2.2 $$LE_{un} = Rn_{un} - H_{un}$$

**Fig. 1:**
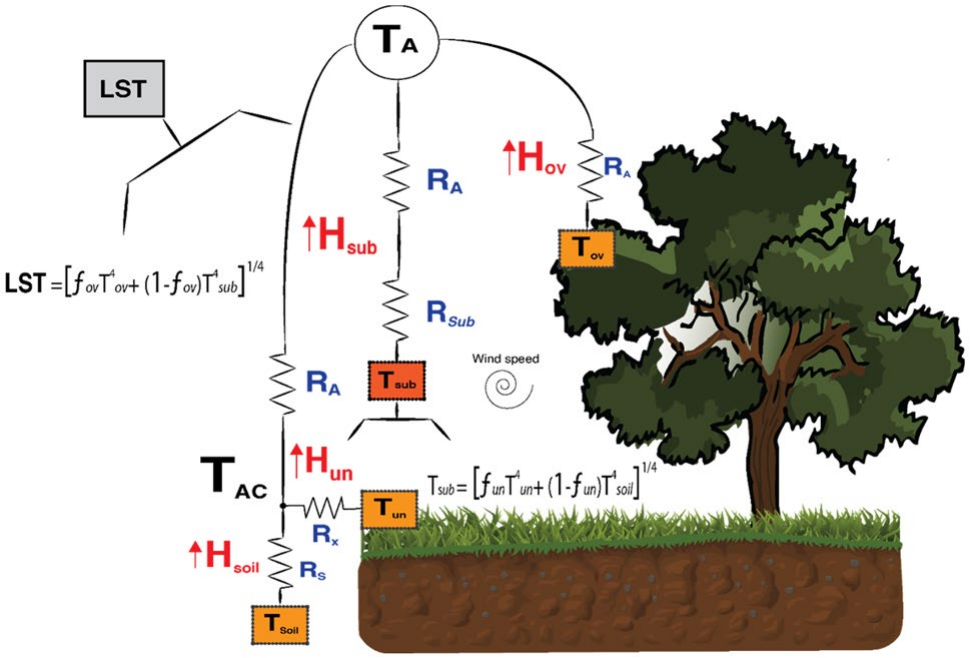
The three- source energy balance (3SEB) model framework (adapted from Burchard-Levine et al. 2022) and its main inputs, including radiometric land surface temperature (LST) and air temperature (TA), and resistance schemes to estimate the sensible heat flux of the overstory vegetation (Hov), understory vegetation (Hun), and soil (Hsoil) sources. Refer to the text for the definition of other symbols

2.3 $$LE_{soil} = Rn_{soil} - H_{soil} - G\,,$$

where LE is the latent heat flux (W m^−2^); Rn is the net radiation (W m^−2^); H is the sensible heat flux (W m^−2^); G is the soil heat flux (W m^−2^). As such, 3SEB directly computes Rn, H, and G and indirectly estimates LE through the residual of the energy balance at each source. The systems of equations are solved within a nested framework where the surface is initially depicted by a parallel overstory-substrate system, and the substrate is subsequently separated into its vegetation (e.g., cover crop) and soil sources through a series (i.e., layered) approach (Fig. 1). For a discussion on the different multi-source representations (e.g., series vs parallel), the reader is referred to Kustas and Norman (1999a, b) and Lhomme et al. (2012). Rn is estimated through the radiative transfer model (RTM) adapted from the model described in Chapter 15 of Campbell and Norman (1998) and detailed in section S1 of supplementary information of Burchard-Levine et al. (2022). G is estimated as the ratio of Rn reaching the soil (i.e., 0.35 $Rn_{soil}$). The resistance-based heat transport equations compute H using Eq. 3.1–3.5

3.1 $$H_{ov} = \frac{{\rho C_{p} \left( {T_{ov} - T_{A} } \right)}}{{R_{A} }}$$

3.2 $$H_{sub} = \frac{{\rho C_{p} \left( {T_{sub} - T_{A} } \right)}}{{R_{A} + R_{sub} }}$$

3.3 $$H_{sub} = H_{un} + H_{soil} = \frac{{\rho C_{p} \left( {T_{AC} - T_{A} } \right)}}{{R_{A} }}$$

3.4 $$H_{un} = \frac{{\rho C_{p} \left( {T_{un} - T_{AC} } \right)}}{{R_{X} }}$$

3.5 $$H_{soil} = \frac{{\rho C_{p} \left( {T_{soil} - T_{AC} } \right)}}{{R_{s} }},$$

where $\rho C_{p}$ is the volumetric heat capacity of air (J m^−3^ K^−1^); $R_{A}$ is the aerodynamic resistance to heat transfer (obtained from Monin–Obukhov Similarity Theory); $R_{Sub}$ is the resistance to heat transfer in the surface boundary layer above substrate layer (s m^−1^); T_AC_, equivalent to the aerodynamic temperature, is the air temperature within the canopy space (K); R_x_ is the bulk canopy resistance to heat transfer (s m^−1^) and R_s_ is the resistance to heat transfer in the boundary layer above the soil surface (s m^−1^).

The Priestley–Taylor (PT) formulation initializes the model at both overstory and understory levels using T_rad_ and T_sub_ as the main boundary conditions

5 $$H_{c} = Rn_{c} - LE_{c} = Rn_{c} \left[ {1 - \alpha_{PT} f_{g,c} \left( {\frac{\Delta }{\Delta + \gamma }} \right)} \right]\,,$$

where $LE_{c}$ (subscript *c* refers to either the understory or overstory vegetation) is the initial canopy transpiration estimate (W m^−2^); $\alpha_{PT}$ is the PT coefficient (default is 1.26) (-), representing only the vegetation canopy component (Agam et al., 2010; Kustas and Anderson, 2009); $f_{g,c}$ is the green vegetation fraction (-); ∆ is the slope of the saturation vapor pressure curve (kPa K^−1^) at air temperature; and γ is the psychrometric constant (kPa K^−1^). An iteration procedure reduces $\alpha_{PT}$, separately for each vegetation layer, until radiometric (Eq. 1) and energy balances (Eq. 2) are conserved under realistic daytime conditions (i.e., no daytime condensation nor negative LE). Refer to Burchard-Levine et al. (2022) or the source code (https://github.com/VicenteBurchard/3SEB) for model details and specifications.

### Study site and data sources

The RIP720 experimental vineyard, located in the Madera County in California’s Central Valley, was the main case study for this work. The study uses hourly meteorological and radiometric data between 2019-01-01 and 2020-12-31, collected as part of the GRAPEX project.

**Fig. 2:**
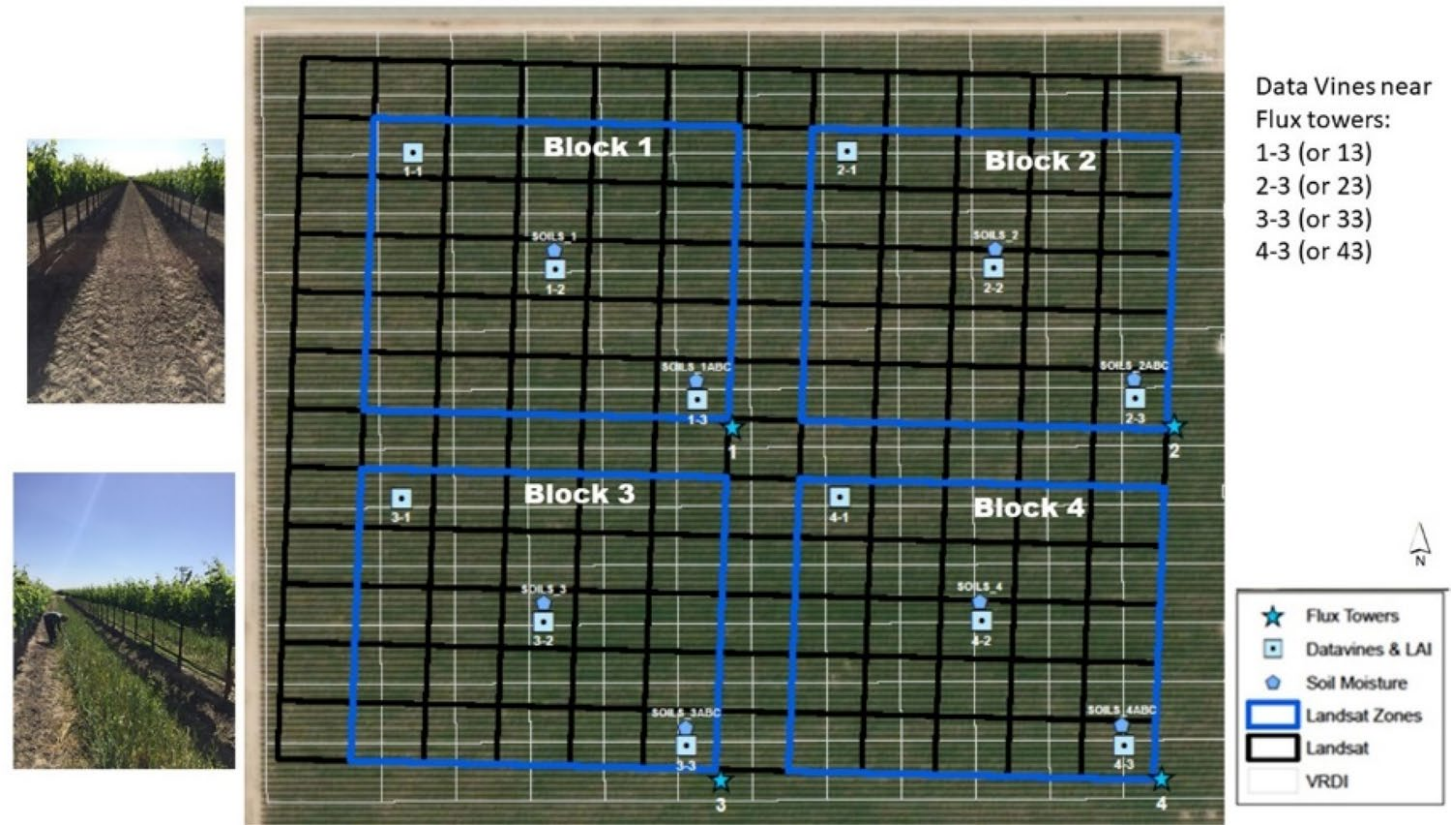
The RIP720 experimental set-up with four blocks, each being sampled continuously by EC towers (blue star points). The white grid is the VRDI system, while the black grid represents the Landsat 30 m resolution pixel coverage over RIP720. Other symbols represent sampling sites for leaf gas exchange, leaf water potential, and leaf area index (blue squares), and profile soil moisture measurements (blue pentagons). In 2019, blocks 1 and 2 had no cover crop in interrow while blocks 3 and 4 had a cover crop present in the interrows

The RIP720 site grows Merlot grapes with an east–west row direction, spaced at 2.74 m, and contains a variable rate drip irrigation (VRDI) system allowing irrigation rates to be prescribed at 30 m resolution. Information pertaining to vineyard geography, vine phenology, vineyard architecture, and agronomic properties of the vineyard block is listed in Table 1. For the GRAPEX project, RIP720 was divided into four blocks (Fig. 2) allowing different irrigation management treatments affecting vine water use and stress for evaluating the satellite-based ET toolkit (Knipper et al. 2019b). Normally, the vineyard is tilled every fall after harvest and a cover crop (CC) is planted. However, in 2019, the CC in blocks 1 and 2 had herbicide sprayed on emergence in January and later mowed in the interrows of the vine foliage, while a CC was planted and allowed to develop as normally in the interrows of blocks 3 and 4. Each block has an eddy-covariance (EC) flux tower equipped to measure the main turbulent (LE and H) and radiometric (Rn and G) fluxes, including meteorological scalars. Along with these continuous measurements, field campaigns in May 2019 measured *in situ* leaf area index (LAI) of both the vine and vine + cover crop systems using the LAI-2200 plant canopy analyzer (LAI-2200) (LICOR Bioscience USA, 2011^1^).

**Table 1:** Information on vineyard characteristics for the four blocks in RIP720 containing the GRAPEX flux towers

Vineyard ID	RIP 720
*Geographic information*	
Vineyard location	Madera County
Height above sea level (m)	61
Topography	Flat
Soil type	Loam/sandy loam
*Vine information*	
Vine variety	Merlot
Year planted	2010
Bud break	March 15—March 29
Flowering/Fruit set	May 1—May 6
Veraison	July 16—July 30
Harvest	Sept 24—Oct 23
*Vineyard architecture*	
Row orientation	East–west
Trellising method	Bilateral cordon (split canopy)
Row width	3.35 m
Planting interval	1.52 m
Vine canopy height (April-Sept)	1.5–2.2 m
*Cover crop information*	
Cover crop type	Perennial grasses
Cover crop width	1.85
*Agronomic and management information*	
Cover crop management	Mowed once or twice in April/May
Irrigation system	Drip irrigation 3L/h (VRDI)
*GRAPEX specific information*	
Date of initial tower deployments	09/04/2018

### Model set-up, inputs, and evaluation

Hourly data between 2019-01-01 and 2020-12-31 were collected to run (i.e., T_rad_ + meteorological) and benchmark (Rn, LE, H, and G) the 3SEB model. TSEB simulations for the same periods were performed and compared against the 3SEB results. In this study, tower-based T_rad_ observations, along with meteorological measurements, forced 3SEB at the tower footprint scale. Meteorological forcing includes incoming shortwave irradiance, air temperature (T_A_), vapor pressure (Ea), and wind speed (u). T_rad_ was retrieved from incoming and outgoing longwave radiation (Lin and Lout, respectively) sampled by a four-component NR01 Net Radiometer, such as in Nieto et al. (2019a).

**Fig. 3:**
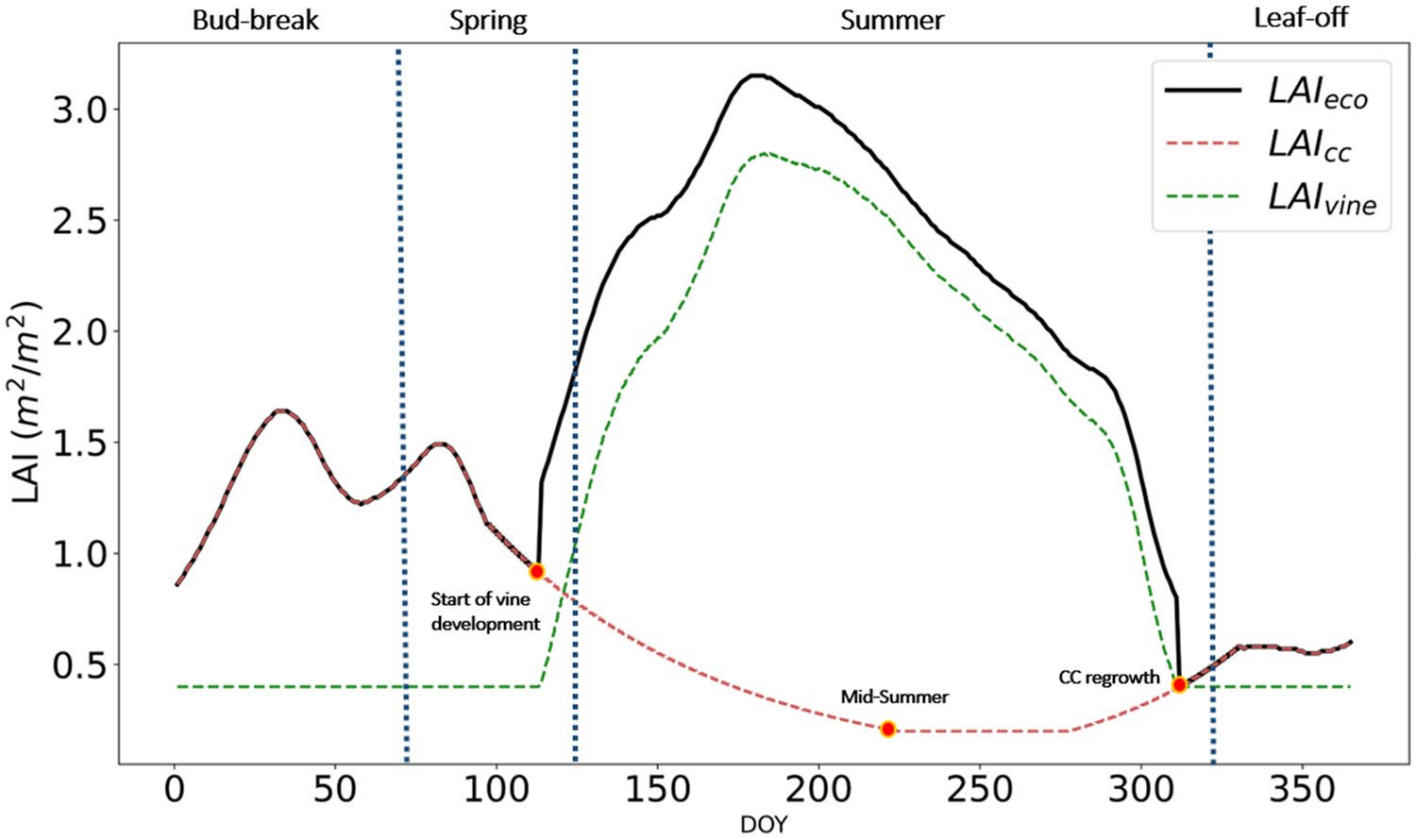
Time series of ecosystem LAI (LAIeco) from satellite imagery (Kang et al., 2022) from block 3 of 2019 decomposed into vine (LAIvine) and cover crop (LAIcc) LAI based on seasonal dynamics

The daily ecosystem LAI were acquired over each tower footprint at 30 m resolution by merging satellite data from the Harmonized Landsat and Sentinel-2 (HLS) surface reflectance and MODIS LAI data product together with in situ LAI measurements (Gao et al. 2012, Kang et al. 2022). The HLS dataset integrates Landsat-8 and Sentinel-2 in a consistent data stream at 30 m resolution (Claverie et al. 2018). The HLS 30 m resolution LAI was generated using a data-driven machine learning approach. The machine learning model (regression tree) was trained using both MODIS LAI samples and ground LAI measurements. Unlike previous LAI versions, this study (LAI version 2.5) used more ground LAI measurements from 2013 to 2020 and multiple vegetation indices to build the LAI model. LAI for HLS dates (3, 4 day repeat) were smoothed and gap-filled using a modified Savitzky–Golay filter to generate daily LAI. The LAI over each tower footprint was extracted from 3 × 3 30 m pixels, thus including vine and cover crop contributions. However, 3SEB needs to explicitly separate the total ecosystem LAI into the contribution of the overstory (i.e., vine) and understory (i.e., cover crop, CC) LAI. The CC normally has a growing phase during early spring and then is mowed in early summer becoming senescent stubble during the main grapevine growing period between May and August. Therefore, the CC LAI (LAI_CC_) was assumed to contribute completely to the total ecosystem LAI (LAI_eco_) before the vine bud-break (~ spring) and after vine leaf-off during the fall. The LAI_CC_ was simulated to exponentially decay from spring until the peak summer period and then exponentially re-grow during the fall period after the vine leaf-off (see Fig. 3). Seasonal transitions dates were corroborated with *in situ* phenocam data. Subsequently, LAI_cc_ was subtracted from the ecosystem LAI to obtain vine LAI (LAI_vine_)

6 $$LAI_{vine} = LAI_{eco} - LAI_{cc} .$$

Indeed, the plant area index (PAI), which incorporates non-leaf biomass (e.g., branches), would be more appropriate, since aerodynamic and radiation transfer is also influenced by non-green elements of vegetation. However, since PAI is difficult to assess through conventional remote-sensing techniques, we use LAI as a proxy. During periods of low LAI, Kobayashi et al. (2012) showed that woody elements play a more prominent role in the radiation transfer through the landscape. Therefore, LAI_vine_ was set to a minimum of 0.4 m^2^/m^2^ during leaf-off periods to account for the effect of branches and woody elements on the radiative and aerodynamic transfer (Burchard-Levine et al. 2022; Ryu et al. 2012). The green fraction (*Fg*) was maintained constant at 1 for vine foliage during leaf-on periods and 0 during leaf-off seasons (with transitions simulated through exponential decay and growth function proportional to LAI). The CC *Fg* was set to 1 during the spring and autumn periods, but simulated to decrease toward 0 during the senescent period using an exponential decay function (similar to LAI_cc_, as shown in Fig. 3). Vine structural parameters, including canopy to row width (*wc*) and canopy height (*hc*), were estimated through empirical relations with LAI (see Table 1 and Nieto et al.2019a). The *wc* and *hc* of the herbaceous CC were assigned as 1 and 0.35 m, respectively.

To account for energy balance closure issues (e.g., Foken et al. 2011), the ‘observed’ H and LE fluxes consist of the averages of three common approaches to account for the lack of energy closure: (1) unclosed, (2) residuals assigned to LE, and (3) the Bowen ratio correction. It is assumed that the ensemble average of the three approaches arrive closest to the actual LE and H observed (Kustas et al., this issue, Bambach et al. this issue). In addition, EC-based ET partitioning (T/ET) estimates were computed through both the Conditional Eddy Covariance (CEC, Zahn et al. 2022) and Modified Relaxed Eddy Accumulation (MREA, Thomas et al. 2008; Zahn et al. 2022) methods. Modeled T/ET were compared against the average of the two EC-based ECC and REA estimates. Modeling performances were quantified with the root-mean-square-deviation (RMSD), mean bias (bias), the Nash–Sutcliffe efficiency index (NSE), and the Pearson’s correlation coefficient (r). Only daytime fluxes were assessed, which is defined here when shortwave irradiance is greater than 100 W/m^2^.

## Results

### 3SEB evaluation

**Fig.4:**
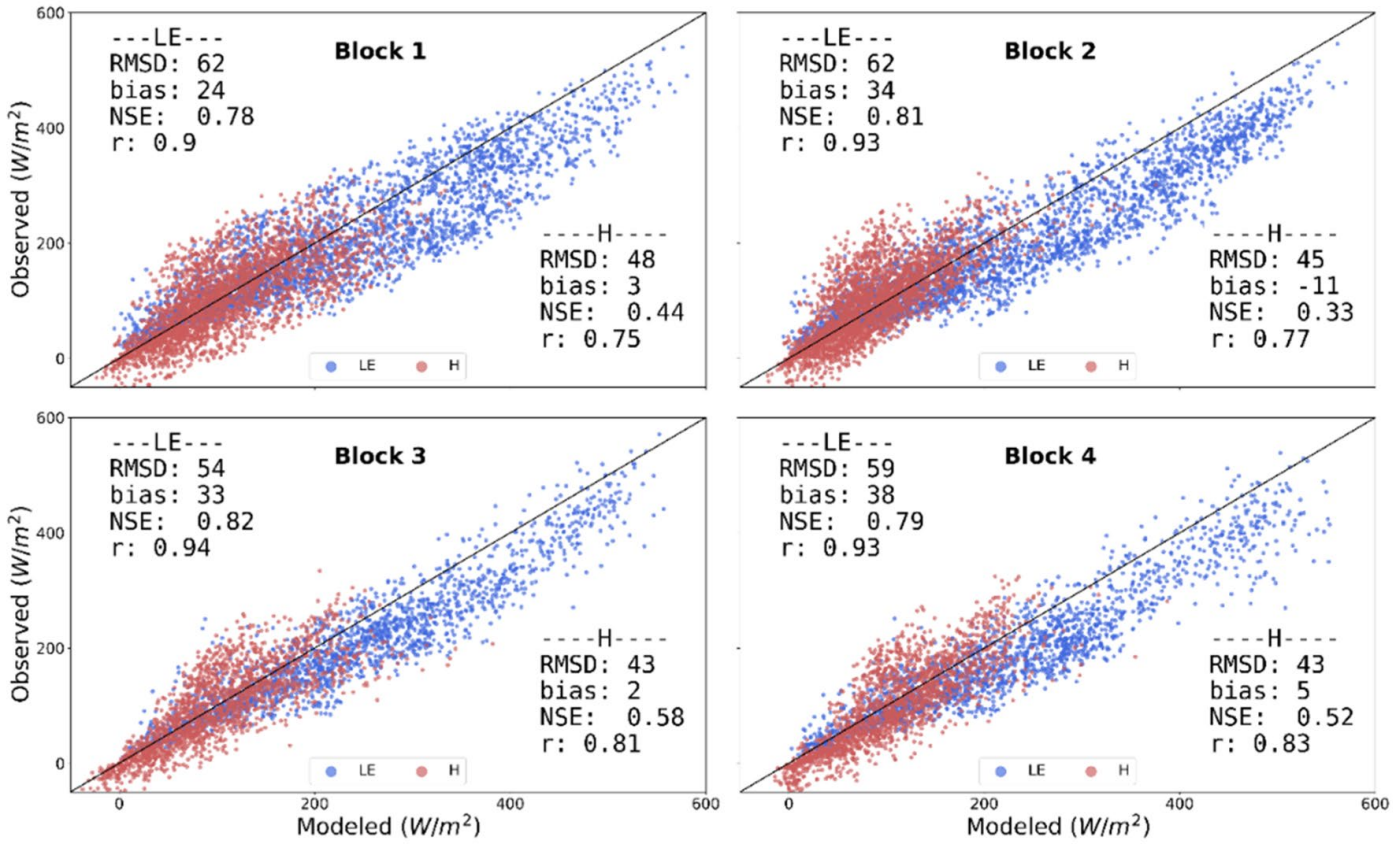
Scatter plots of 3SEB estimated daytime hourly LE (blue) and H (red) in 2019 versus those measured from the EC flux towers within the four blocks in RIP720. The black line represents the 1:1 line

**Fig. 5:**
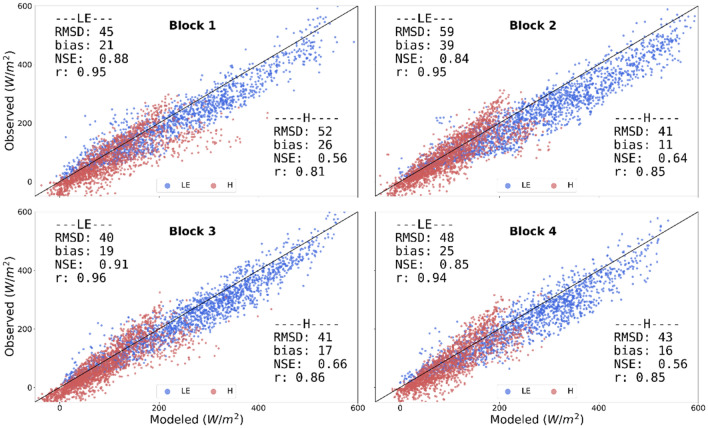
Scatter plots of 3SEB estimated daytime hourly LE (blue) and H (red) in 2020 versus those measured from the EC flux towers within the four blocks in RIP720. The black line represents the 1:1 line

3SEB achieved low daytime hourly LE and H errors (RMSD < 65 W/m^2^) over the four blocks in RIP720 for the years 2019 (Fig. 4) and 2020 (Fig. 5). In general, LE tended to have slightly larger errors compared to H, where it was generally slightly overestimated (LE Bias ~ 20–30 W/m^2^). The modeled LE had the largest errors in blocks 1 and 2 in 2019 (LE RMSD ~ 60 W/m^2^), the period when there was no CC. These larger errors were related to the overestimation of available energy (see Rn and G error statistics in Table 2), since H errors maintained to within similar magnitudes as other blocks/years (H RMSD ~ 40 W/m^2^).

**Table 2:** Model performance indicators (RMSD, bias, NSE and r) of daytime LE, H, Rn, and G with TSEB and 3SEB for the different seasons of blocks 1 and 2 in RIP720 for 2019 and 2020

Sites	Season vine phenological stage	Flux	Model	RMSD (W/m^2^)	r (−)	Bias (W/m^2^)	NSE (−)
Blocks 1 + 2	Winter to Bud-Break (DOY ≤ 90)	**LE**	TSEB	**36**	0.77	**4**	**0.57**
			**3SEB**	41	0.77	12	0.55
		**H**	TSEB	36	0.82	− 6	0.65
			**3SEB**	**30**	**0.86**	− **1**	**0.72**
		**Rn**	TSEB	41	0.994	− 38	0.91
			**3SEB**	**39**	0.991	− **34**	**0.92**
		**G**	TSEB	**28**	**0.84**	− **14**	**0.36**
			**3SEB**	37	0.73	− 21	0.12
	Spring/bloom and berry development (DOY ~ 90 to ~ 150)	**LE**	TSEB	**52**	0.80	**− 2**	0.63
			**3SEB**	54	**0.87**	**17**	**0.71**
		**H**	TSEB	64	0.71	22	0.45
			**3SEB**	**52**	**0.77**	**19**	**0.51**
		**Rn**	TSEB	**15**	**0.998**	− **1**	**0.99**
			**3SEB**	17	0.996	**4**	0.99
		**G**	TSEB	25	0.71	− 7	0.21
			**3SEB**	**24**	**0.78**	− 11	0.24
	Summer to Fall/Grape bunch closure through Veraison, Harvest and Senescence (DOY ~ 150 to ~ 330)	**LE**	TSEB	**63**	**0.91**	**34**	**0.78**
			**3SEB**	64	0.91	39	0.76
		**H**	TSEB	50	0.75	− 3	0.17
			**3SEB**	**49**	**0.76**	**3**	**0.24**
		**Rn**	TSEB	**15**	**0.998**	− **2**	0.99
			**3SEB**	17	0.996	3	0.99
		**G**	TSEB	21	0.73	3	0.38
			**3SEB**	18	0.79	− 1	0.47
	Fall-to-Winter Leaf-off (DOY ~ 330 to 365)	**LE**	TSEB	**16**	**0.93**	**0**	**0.81**
			**3SEB**	**23**	0.93	11	0.74
		**H**	TSEB	30	**0.91**	− 19	0.47
			**3SEB**	**21**	0.90	− 2	0.72
		**Rn**	TSEB	38	0.999	− 36	0.82
			**3SEB**	**24**	0.999	**− 22**	**0.92**
		**G**	TSEB	**11**	**0.90**	**− 5**	**0.64**
			**3SEB**	21	0.89	− 17	− 0.06
	All seasons	**LE**	TSEB	**57**	0.93	22	0.84
			**3SEB**	59	**0.93**	30	0.82
		**H**	TSEB	50	0.75	1	0.45
			**3SEB**	**47**	**0.78**	5	**0.48**
		**Rn**	TSEB	**23**	**0.995**	− 11	0.98
			**3SEB**	24	0.993	− **6**	0.98
		**G**	TSEB	**27**	0.77	− **9**	**0.37**
			**3SEB**	30	**0.78**	− 14	0.32

The best resulting model indicator for each flux between TSEB and 3SEB is highlighted in bold

### TSEB and 3SEB comparison

The TSEB model performed similarly well in all four blocks in RIP720, achieving errors statistics within comparable magnitudes to the 3SEB simulations (Tables 2, 3). Overall, 3SEB only slightly improved over TSEB, with LE and H RMSD decreasing 55 to 52 W/m^2^ and 47 to 43 W/m^2^, respectively, aggregating for all blocks and years. 3SEB improved flux simulations more significantly in blocks 3 and 4, which had the presence of herbaceous CC for both 2019 and 2020 years. For blocks 3 and 4, LE RMSD decreased from 56 to 51 W/m^2^ and the H NSE increased from 0.43 to 0.58.

**Table 3:** Model performance indicators (RMSD, bias, NSE, and r) of daytime LE, H, Rn, and G with TSEB and 3SEB for the different seasons of blocks 3 and 4 in RIP720 for 2019 and 2020

Sites	Season vine phenological stage	Flux	Model	RMSD (W/m^2^)	r (−)	Bias (W/m^2^)	NSE (−)
Blocks 3 + 4	Winter to Bud-Break (DOY ≤ 90)	**LE**	TSEB	37	0.79	2	0.61
			**3SEB**	**35**	**0.89**	17	**0.75**
		**H**	TSEB	**37**	0.85	**8**	**0.71**
			**3SEB**	42	**0.90**	28	**0.69**
		**Rn**	TSEB	34	0.997	− 33	0.94
			**3SEB**	**20**	0.997	− **17**	**0.98**
		**G**	TSEB	**30**	0.81	**− 21**	**0.18**
			**3SEB**	46	**0.86**	− 39	− 0.22
	Spring/bloom and berry development (DOY ~ 90 to ~ 150)	**LE**	TSEB	52	0.81	− **8**	0.64
			**3SEB**	**51**	**0.88**	24	**0.71**
		**H**	TSEB	61	0.68	**8**	0.39
			**3SEB**	**48**	**0.81**	22	**0.50**
		**Rn**	TSEB	22	0.999	− 21	0.98
			**3SEB**	**8**	0.999	**− 3**	**0.99**
		**G**	TSEB	37	0.62	**8**	**0.32**
			**3SEB**	**35**	**0.63**	− 19	0.06
	Summer to Fall/Grape bunch closure through Veraison, Harvest and Senescence (DOY ~ 150 to ~ 330)	**LE**	TSEB	62	**0.94**	38	**0.82**
			**3SEB**	**54**	0.93	**33**	0.81
		**H**	TSEB	45	**0.91**	− 21	0.03
			**3SEB**	**41**	0.86	**2**	**0.40**
		**Rn**	TSEB	**14**	**0.998**	**− 5**	**0.99**
			**3SEB**	17	0.997	9	0.99
		**G**	TSEB	21	0.73	3	0.38
			**3SEB**	**18**	**0.79**	− **1**	**0.47**
	Fall-to-Winter Leaf-off (DOY ~ 330 to 365)	**LE**	TSEB	**19**	**0.91**	**2**	**0.81**
			**3SEB**	28	0.89	12	0.71
		**H**	TSEB	29	**0.91**	− 19	0.50
			**3SEB**	**22**	0.88	**1**	**0.74**
		**Rn**	TSEB	42	**0.999**	− 40	0.84
			**3SEB**	**27**	**0.999**	− **26**	**0.92**
		**G**	TSEB	**10**	**0.92**	− **4**	**0.79**
			**3SEB**	22	0.90	− 18	0.02
	All seasons	**LE**	TSEB	56	**0.95**	**25**	**0.86**
			**3SEB**	**51**	0.94	28	0.84
		**H**	TSEB	46	0.78	− 12	0.43
			**3SEB**	**42**	**0.84**	**10**	**0.58**
		**Rn**	TSEB	21	0.997	− 13	0.99
			**3SEB**	**16**	**0.997**	**2**	**0.99**
		**G**	TSEB	**26**	**0.69**	− **1**	**0.33**
			**3SEB**	28	0.65	− 11	0.03

The best resulting model indicator for each flux between TSEB and 3SEB is highlighted in bold

Most importantly, the differences between 3SEB and TSEB were most apparent during the spring period, with the H RMSD decreasing from 64 to 52 W/m^2^ and from 61 to 48 W/m^2^ in blocks 1 + 2 and blocks 3 + 4, respectively. During this period, the LE error statistics did not improve as significantly, and were very similar between TSEB and 3SEB (Tables 2, 3) due to uncertainties in the available energy, particularly underestimating G. Annex 1 shows results when both TSEB and 3SEB were forced with observed G, thus limiting the uncertainty of the AE. When forcing G measurements into the models, LE estimations from 3SEB improved similarly to H and showed overall improvement over TSEB (RMSD decreased from 55 to 46 W/m^2^), particularly during the spring (RMSD decreased from 81 to 54 W/m^2^).

During periods of low grapevine foliage (i.e., winter), LE and H error statistics were generally low and similar between 3SEB and TSEB. However, 3SEB tended to simulate Rn with less bias than TSEB during these periods, while simultaneously having more uncertainties in G. For example, prior to bud-break, the 3SEB Rn bias was − 17 W/m^2^ compared to TSEB’s − 33 W/m^2^, but bias in simulated G increased from − 21 to − 33 W/m^2^ during this period with 3SEB.

**Fig. 6:**
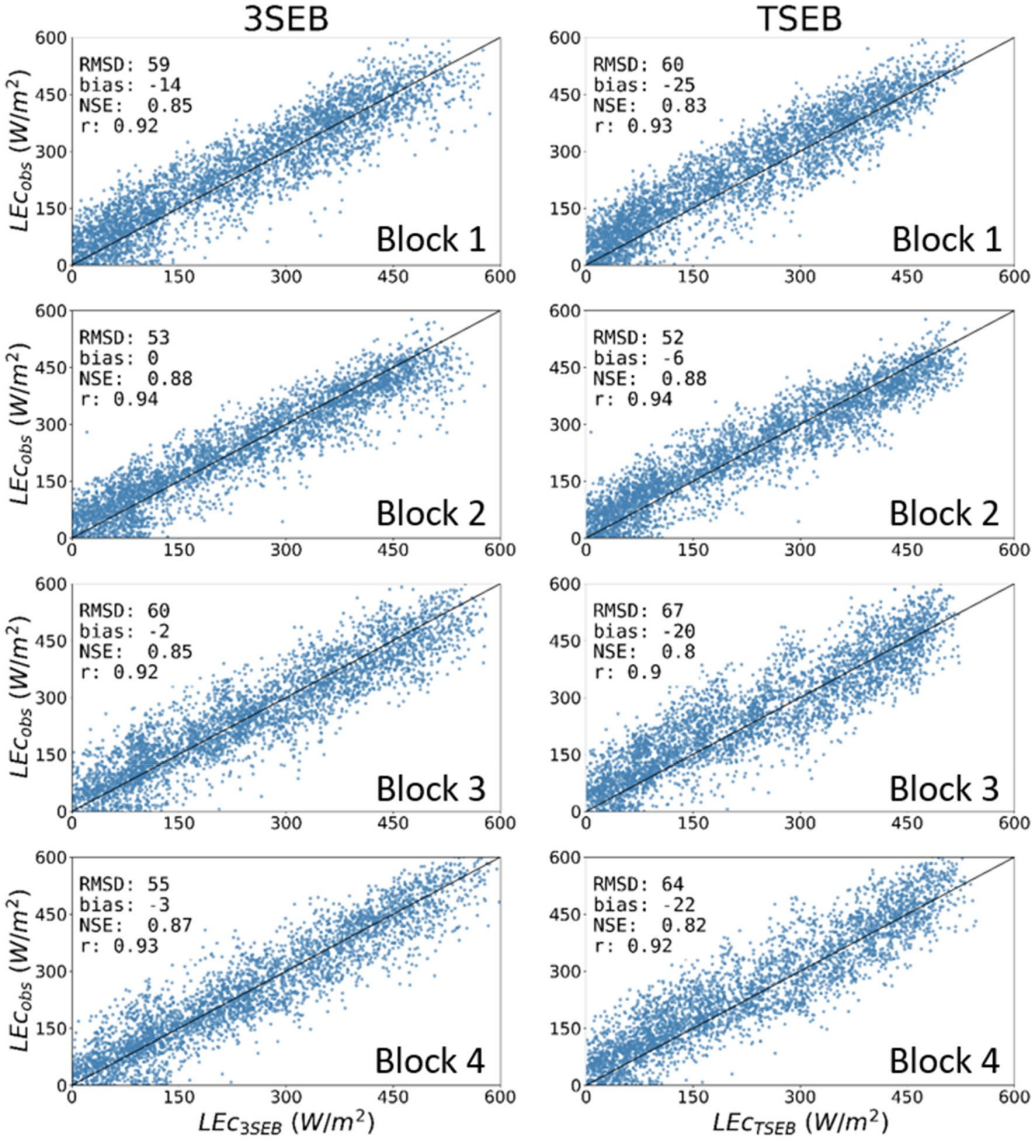
Scatter plots of 3SEB (left column) and TSEB (right column) estimated daytime hourly canopy (vine + CC) LE (LEc) (blue) versus those measured from the EC flux towers using the CEC and REA approach between 2018 and 2019. The black line represents the 1:1 line

TSEB generally slightly underestimated transpiration (vine + CC) or LEc compared to the EC-based approach (Fig. 6). For all blocks, there was less systematic bias with 3SEB (~ − 5 W/m^2^) compared to TSEB (> − 20 W/m^2^). Notably, 3SEB showed greater improvement in blocks 3 and 4, where LEc RMSD decreased from roughly 66 to 58 W/m^2^ when applying 3SEB compared to TSEB. In blocks 1 and 2, LEc error statistics were very similar between TSEB and 3SEB (Fig. 6).

### Seasonal ET partitioning

**Fig. 7:**
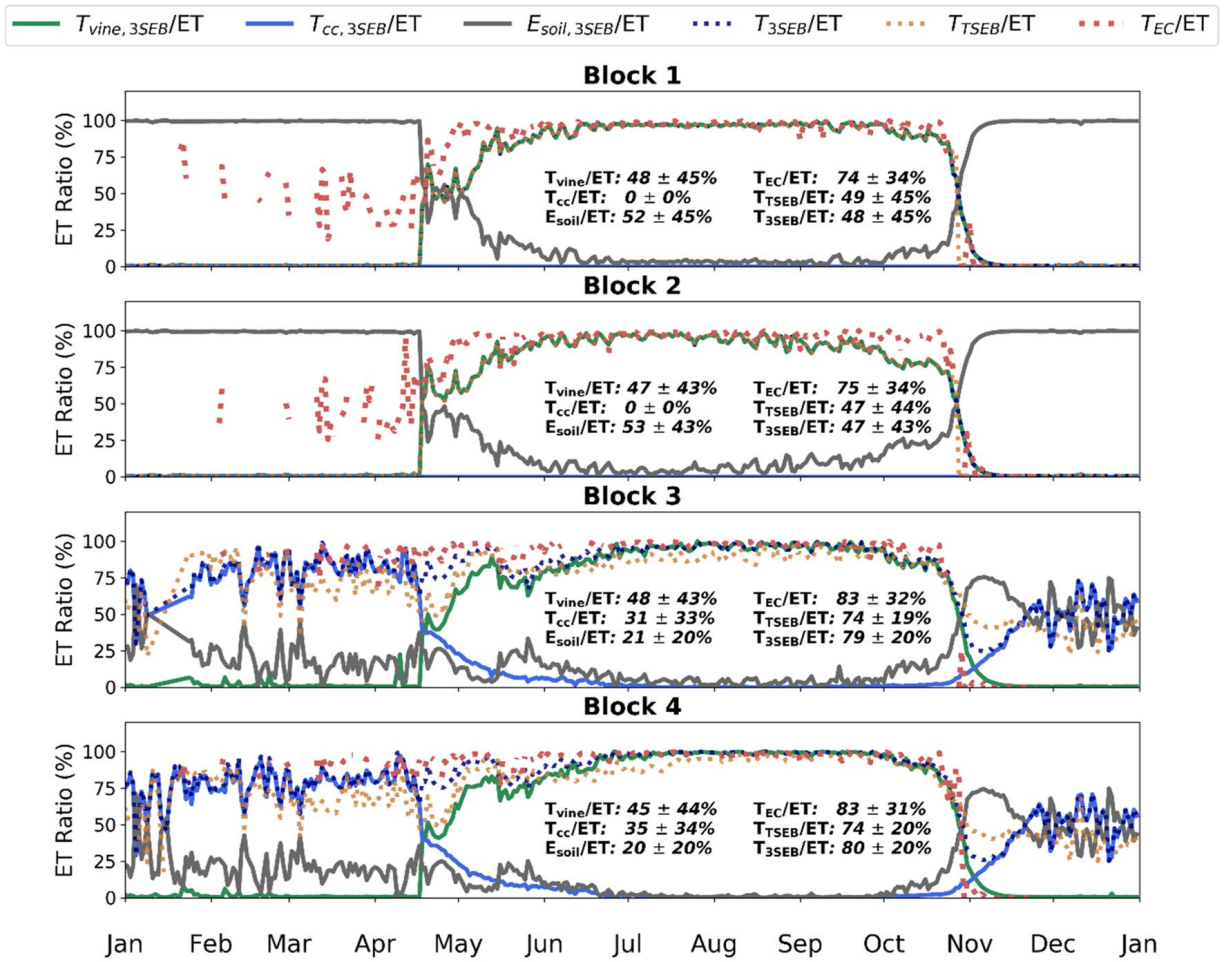
Average daily seasonal ET partitioning into vine (green), cover crop (blue), and soil (gray) as a percentage of total surface ET by 3SEB during 2019 for blocks 1–4 in RIP720. Average daily transpiration partitioning time series estimated through 3SEB (vine + cover crop, dotted blue), TSEB (dotted orange), and through eddy-covariance methods (dotted red) are also shown for each block. During 2019, an experiment was conducted with no cover crop for blocks 1 and 2, while the cover crop was maintained in blocks 3 and 4. The percentage values of T/ET listed in the figure represent the annual daily average and the corresponding standard deviation

Figure 7 shows the 2019 daytime average time series of ET decomposed into the different landscape sources, such as vine transpiration (T_vine_), cover crop transpiration (T_cc_), and soil evaporation (E_soil_). During 2019, the herbaceous CC was treated with herbicides and later mowed in blocks 1 and 2, as shown in Fig. 7, with no modeled T_cc_ in blocks 1 and 2. T_vine_ dominates the surface ET during the spring and summer periods in all four blocks of the RIP720 site. Mean T_vine_/ET is roughly 50% in all blocks. T_cc_ in blocks 3 and 4 was slightly greater than E_soil_ (~ 30% and ~ 20%, respectively). Mean annual surface transpiration (T_vine_ + T_cc_) was roughly 80% in blocks 3 and 4, while modeled T/ET was significantly less in blocks 1 and 2 (~ 50%), with most of the proportion dedicated to T_cc_ being taken up by E_soil_.

Overall, 3SEB’s annual T/ET estimate (~ 80%) was of similar magnitude to the EC estimate transpiration (T_EC_/ET; 83%) in blocks 3 and 4. In general, TSEB slightly underestimated T/ET (~ 74%) compared to the EC and 3SEB approach, even though TSEB followed very similar patterns to 3SEB, albeit being slightly lower in magnitude, in blocks 3 and 4. The largest differences occur during the transitional periods when the CC senesces, and the grapevine begins to grow (April–May) and vice-versa (October–November). TSEB shows a momentary dip in T/ET toward the end of April, while the 3SEB, and EC-based, T/ET maintain high levels (> 75%). Additionally, modeled T/ET increases again toward the end of the year when the CC regrows; however, a lack of observed T_EC_/ET during that period does not allow us to confirm if this was also captured by this EC-based method. In blocks 1 and 2, 3SEB and TSEB T/ET are essentially identical as there was no CC in these blocks. The overall modeled T/ET (50%) was substantially less than T_EC_/ET (75%). Prior to the vine bud-break, the EC-based method maintains high T/ET rates (> 50%), while modeled T/ET is roughly zero during this period as the CC was not present (i.e., herbicide application and mowing), and consequently, the understory is simulated as bare soil by the 3SEB model. It may be that the high T/ET rates computed by the EC partitioning techniques are caused by transpiration sources affecting the sensors from upwind neighboring almond orchards (~ 300 m away), since, particularly in the period February through March prior to vine bud-break, the almonds flower and start actively transpiring. Combined with more stable conditions, this advection would cause a much larger source area affecting the EC measurements.

## Discussion

Using T_rad_ measurements from tower-based longwave radiometers, the 3SEB model accurately simulated LE and related energy fluxes for a clumped and complex vineyard landscape. The 3SEB overall modeled LE and H RMSD, over all four blocks in 2019 and 2020, was 52 and 43 W/m^2^, respectively. The higher LE errors, as compared to H, were due to increased uncertainties in the available energy. This was most notable for blocks 1 and 2 during 2019 (Fig. 4 and Table 1) when the CC was removed. The larger LE errors were driven by underestimations in G in these blocks, since LE is estimated as the residual of the energy balance. The removal of the CC likely caused more irradiance to reach the soil, including more energy retention due to changes in albedo. Although Rn was relatively well modeled, albeit slightly underestimated (Rn Bias in blocks 1 and 2 was − 6 W/m^2^), the Rn transmitted and reaching the soil layer may have been underestimated within 3SEB. However, it is difficult to evaluate this without direct observations. More complex and/or three-dimensional RTM methods, such as those discussed in Parry et al. (2019) for vineyards, may enlighten and provide an indicator of the available energy transmitted through the canopies. The CC removal likely affected the amount of water retained in the soil, causing higher soil temperatures and changes to the thermal diffusivity of soils inducing more heat conduction (Li et al. 2014). Annex 1 shows model results when 3SEB (and TSEB) were forced with observed G (instead of estimated through the ratio approach). As shown in Table S1, when forced with observed G, 3SEB modeled LE RMSD and bias in block 1 and 2 decreased from 59 to 48 W/m^2^ and 30 to − 12 W/m^2^, respectively. Similar improvements were found in blocks 3 and 4 (Table S2) where LE RMSD and bias decreased from 45 to 43 W/m^2^ and 19 to 1 W/m^2^, respectively. As shown, the uncertainties related to AE are directly transposed into uncertainties in LE within energy balance approaches. More investigation is needed to further improve G modeling in vineyards, especially those without a CC. This may include incorporating different approaches to estimate G, which more dynamically assess the temporal behavior of G, similar to the semi-empirical approach developed by Nieto et al (2019b). This is especially important in semi-arid and arid landscapes as G plays a more prominent role in the energy balance of these regions (Heusinkveld et al. 2004; Li et al. 2014).

Overall, errors statistics slightly improved with 3SEB compared against TSEB simulations in all four blocks of RIP720 (Tables 2, 3). The 3SEB improvement was most notable when CC was not removed from the interrow (i.e., blocks 3 + 4 and blocks 1 + 2 in 2020), especially during the spring season and when the models were forced with observed G (Annex 1). During these transitional seasons, more mixing is observed between vine foliage and the CC vegetation. During the spring, the vine buds break and begin to develop, co-dominating with the active CC. In the summer, the vine foliage starts dominating the land surface, while the CC senesces with significant mixing still occurring during the early summer. The 3SEB model structure is more suited for these mixed conditions, as it inherently differentiates these vastly different vegetation layers within its framework. Using bulk vegetation and structural parameters when both vegetation types are active, such as in TSEB, may cause larger uncertainties, with past studies showing the non-linear relationship between vegetation structural parameters and heat fluxes (Burchard-Levine et al. 2022; Kustas et al. 2004; Moran et al. 1997). Flux estimations with TSEB and 3SEB performed similarly before the bud-break and after vine harvest (i.e., winter–autumn).

However, it is noteworthy that Rn simulations improved considerably with 3SEB during the bud-break and leaf-off periods. For example, in blocks 3 and 4, Rn RMSD decreased during the bud-break and leaf-off periods from 34 to 18 W/m^2^ and 41 to 24 W/m^2^, respectively. In fact, modeled turbulent fluxes (LE and H) also improved with 3SEB when forced with observed G (Annex 1) during these seasonal periods, likely driven by improved available energy estimations. 3SEB applies an adapted version of the RTM described in Campbell and Norman (1998), incorporating an additional vegetation canopy and the effect of the tall overstory shadow over the understory (Burchard-Levine et al. 2022). During leaf-off, the grapevine is not photosynthetically active (i.e., *Fg* is zero), but the woody elements nonetheless affect the radiation and aerodynamic transmission. As found in Kobayashi et al. (2012), branches and other wooded structures within an oak woodland absorbed a significant portion of shortwave radiation during periods of low LAI. During grapevine leaf-off, 3SEB maintains a minimum LAI_vine_ of 0.4, as suggested by Ryu et al. (2012) to account for woody material for deciduous Oaks. This may contribute to 3SEB’s improved Rn estimations, capable of incorporating LAI_CC_ and woody elements of grapevine, as compared to TSEB, which only simulates a singular vegetation canopy.

Besides improving total landscape fluxes, 3SEB internally separates the flux contribution from the vine, CC and soil. When the CC is present, 3SEB estimated an annual T/ET of about ~ 80%, which was only slightly less compared to the EC-based partitioning approaches (~ 83%) that are also subject to their own uncertainties. This slight underestimation was also supported when comparing the LEc at the hourly scale (Fig. 6), where 3SEB’s LEc had an RMSD and bias of ~ 60 and ~ − 5 W/m^2^, respectively, compared to the EC-based approach. 3SEB-based LEc also achieved less bias compared to TSEB (i.e., bias ~ −20 W/m^2^), especially when the herbaceous CC was allowed to grow in the interrow. The direct accounting for the CC effect on the energy balance within 3SEB likely contributed to this slight improvement. Most studies assume the CC contribution to total water fluxes is negligible (Nieto et al. 2019b); however, the 3SEB modeling results indicate an average ~ 30% contribution of the herbaceous understory to daily ET in these semi-arid conditions, mostly dominating when the grapevine is not photosynthetically active. In annual volumetric terms, modeled ET from the interrow (i.e., CC transpiration + soil evaporation) corresponded to roughly 255 mm/year in 2019 for blocks 3 and 4. This was very similar to the estimated ~ 250 mm/year from the interrow (i.e., soil evaporation) of blocks 1 and 2, which had the herbaceous CC removed.

At the daily scale, we observed some seasonal differences between the modeled T/ET (T_3SEB_/ET) and EC-based approach (T_EC_/ET). As shown in Fig. 7 in blocks 3 and 4, T_EC_/ET maintains close to unity throughout the year, until decreasing quickly in late October–November during the vine senescence. T_3SEB_/ET shows more variability, achieving around 75–80% during pre-bud-break and spring periods before reaching near unity during the summer period when the vine is fully developed. Additionally, when there was no cover crop (i.e., block 1 and 2 in 2019), T_3SEB_/ET was ~ 0% prior to the development of the vine foliage as 3SEB considers the surface as essentially a bare soil (i.e., vine *Fg* is 0 during this period). However, T_EC_/ET was ~ 50% during this period, even though the CC was sprayed with herbicide on emergence in January. Phenocam images show a small presence and growth of the herbaceous cover in these blocks before it was mowed in mid-February, which would not have been accounted by 3SEB with the current model set-up. There may also be an issue of the eddy-covariance measurements being affected by the upwind almond orchards in February and March when the almond trees flower and begin transpiring prior to vineyard, which bud-break in mid-to-late-March. Different T/ET partitioning methods (i.e., Nelson et al. 2020; Stoy et al. 2019) should also be further explored to enhance the comparison and validation of the ET partitioning from 3SEB.

As discussed in Kustas et al. (2019), the T/ET partitioning in TSEB (and 3SEB) is strongly controlled by LAI and *Fg*; therefore, it remains to be understood whether these model deviations are due to inaccuracies from the model inputs, or whether the model structure is not fully incorporating certain controlling factors of T/ET. For example, it has been shown that trees have a strong physiological and stomatal control on ET in semi-arid regions, notably in conditions of high vapor pressure deficit (VPD) (Pérez-Priego et al. 2010; Villalobos et al. 2012), including within vineyards (Collins et al. 2010; Rogiers et al. 2012). These effects may not be fully captured by the Priestley–Taylor (PT) initialization in both TSEB and 3SEB, with some studies suggesting to lower the α PT coefficient in TSEB for semi-arid conditions (e.g., Andreu et al., 2018). As mentioned in Burchard-Levine et al. (2022), investigating alternative initialization formulations in TSEB and 3SEB, such as using a Penman–Monteith type equation with a stomatal conductance model, may be a way forward to improve T/ET estimates (Kustas et al. 2022).

Reliable estimates of vine transpiration are highly valuable to determine water stress and to adequately manage irrigation to improve water use efficiency and obtain high-quality grape yields. 3SEB has the advantage of directly accounting for both CC and vine, which gives it high potential to disentangle total ET into that directly contributed by the vine foliage. In addition, the 3SEB model structure remains relatively simple, not requiring large parameterization, and therefore could be easily applicable for different sites. A key limitation remains in the accurate and operational separation of LAI_eco_ into LAI_vine_ and LAI_cc_. This study used prior knowledge of key seasonal dates to separate LAI_eco_ into the different vegetation components, but other techniques should be investigated, such as spectral unmixing (e.g., Meyer and Okin 2015), to more dynamically partitioning LAI_eco_. Alternatively, Planet data at 3 m resolution may also help to detect cover crops from vine development in the early season prior to and for a period after bud-break (Roy et al. 2021). Although this study was performed at the tower-level, 3SEB may be implemented with airborne or satellite (Burchard-Levine et al. 2022) images. This has the potential to obtain spatially distributed vine water stress indices, which may complement irrigation scheduling to improve vineyard water and yield management.

## Conclusions

This study explored the use of a simplified remote-sensing-based three-source energy balance model (3SEB) to estimate ET in clumped vineyard landscape in semi-arid California. Model simulations were performed in four blocks in the RIP720 experimental vineyard, where two blocks had the herbaceous cover crop (CC) removed during the 2019 growing period. 3SEB achieved high accuracy in simulating ET and related energy fluxes (LE RMSD ~ 50 W/m^2^), slightly improving over two-source modeling (i.e., TSEB). 3SEB’s improvement over TSEB was more significant during the spring and early summer seasons, where there is a larger degree of mixing between vine foliage and herbaceous CC. Estimated LE from 3SEB did not differ considerably from TSEB during vine leaf-off periods (e.g., autumn–winter). However, 3SEB Rn was more accurately simulated during this period of low vine foliage, suggesting that it better incorporated the effect of woody elements on radiation transmission. 3SEB simulations forced with observed G showed that LE and H improved more significantly for all seasons compared to TSEB, also during periods of low vine foliage, suggesting that accurate G retrieval methods are important to achieve reliable ET estimates.

3SEB additionally allows for a framework to separate the water flux contributions from the grapevine and CC. The annual transpiration contribution of the CC was not negligible (contributing on average to ~ 30% of daily ET), mainly concentrating during grapevine leaf-off periods (autumn–winter). This has the potential to improve water accounting at both vineyard and regional scales. In addition, this can improve grapevine water stress indices, through the separation of the vine transpiration, which would better support irrigation managers for water resource management and grape quality control. Modeled T/ET was largely in line with an EC-based approach, being comparatively only slightly underestimated. Additional datasets, such as EC measurements of the CC understory, are needed to validate the modeled T/ET and investigate whether uncertainties are related to uncertainties in model inputs, model structure, or other sources.

These results suggest the promising applicability of the 3SEB model to monitor vineyards and develop robust grapevine water stress indices to support irrigation scheduling and enhance agronomic water use efficiency. The improvement in T/ET partitioning for both understory and overstory sources, combined with spatial remote-sensing imaging, can be of utility to support grapevine irrigation and production from field to regional scales.

## Supplementary Information

Below is the link to the electronic supplementary material.

Supplementary file1 (DOCX 26 KB)

## Footnotes

1 The use of trade, firm, or corporation names in this article is for the information and convenience of the reader. Such use does not constitute official endorsement or approval by the US Department of Agriculture nor the Agricultural Research Service of any product or service to the exclusion of others that may be suitable.
