# Supplementary material for: Application of a remote-sensing three-source energy balance model to improve evapotranspiration partitioning in vineyards
Source: Irrig Sci. 2022 Apr 5;40(4-5):593–608. doi: 10.1007/s00271-022-00787-x (PMC9509310; doi:10.1007/s00271-022-00787-x)
Supplement: Supplementary file 1 — Supplementary file1 (DOCX 26 KB) [file 271_2022_787_MOESM1_ESM.docx]

**Annex 1**

**Table S1.** Model performance indicators (RMSD, bias, NSE and r) of daytime LE, H, and Rn with TSEB and 3SEB (both forced with observed G) for the different seasons of blocks 1 and 2 in RIP720 for 2019 and 2020. The best resulting model indicator for each flux between TSEB and 3SEB are highlighted in orange.

| **Sites** | **Season** | **Flux** | **Model** | **RMSD (W/m^2^)** | **r (-)** | **Bias (W/m^2^)** | **NSE (-)** |
| --- | --- | --- | --- | --- | --- | --- | --- |
|  | **Vine Phenological stage** |  |  |  |  |  |  |
| **Blocks 1+2** | **Winter to Bud-Break (DOY ≤ 90 )** | **LE** | TSEB | 46 | 0.82 | -33 | 0.39 |
|  |  |  | **3SEB** | **40** | **0.94** | -34 | **0.56** |
|  |  | **H** | TSEB | 37 | 0.80 | -7 | 0.63 |
|  |  |  | **3SEB** | **25** | **0.91** | **-7** | **0.78** |
|  |  | **Rn** | TSEB | **42** | 0.995 | **-40** | **0.90** |
|  |  |  | **3SEB** | 44 | **0.997** | -41 | 0.90 |
|  | **Spring/bloom and berry development (DOY ~90 to ~150)** | **LE** | TSEB | 87 | 0.61 | -48 | 0.21 |
|  |  |  | **3SEB** | **51** | **0.85** | **-22** | **0.68** |
|  |  | **H** | TSEB | 78 | 0.65 | 28 | 0.37 |
|  |  |  | **3SEB** | **46** | **0.82** | **7** | **0.64** |
|  |  | **Rn** | TSEB | 24 | **0.997** | -20 | 0.98 |
|  |  |  | **3SEB** | 24 | 0.993 | **-15** | 0.98 |
|  | **Summer to Fall/Grape bunch closure through Veraison, Harvest and Senescence (DOY ~150 to ~330)** | **LE** | TSEB | 50 | **0.92** | **-5** | **0.83** |
|  |  |  | **3SEB** | **49** | 0.91 | -6 | 0.82 |
|  |  | **H** | TSEB | 54 | 0.71 | **3** | 0.20 |
|  |  |  | **3SEB** | **53** | **0.72** | 8 | **0.26** |
|  |  | **Rn** | TSEB | **15** | **0.998** | **-2** | 0.99 |
|  |  |  | **3SEB** | 17 | 0.996 | 3 | 0.99 |
|  | **Fall to Winter Leaf-off (DOY ~330 to 365)** | **LE** | TSEB | 26 | 0.86 | -17 | 0.56 |
|  |  |  | **3SEB** | **23** | **0.93** | **-16** | **0.72** |
|  |  | **H** | TSEB | 30 | 0.91 | -20 | **0.50** |
|  |  |  | **3SEB** | **21** | **0.88** | **-3** | 0.40 |
|  |  | **Rn** | TSEB | 38 | 0.999 | -37 | 0.82 |
|  |  |  | **3SEB** | **21** | **0.999** | **-19** | **0.94** |
|  | **All seasons** | **LE** | TSEB | 56 | 0.92 | **-17** | 0.83 |
|  |  |  | **3SEB** | **48** | **0.93** | -12 | **0.86** |
|  |  | **H** | TSEB | 56 | 0.71 | **5** | 0.42 |
|  |  |  | **3SEB** | **49** | **0.77** | 6 | **0.47** |
|  |  | **Rn** | TSEB | 24 | **0.995** | -11 | 0.98 |
|  |  |  | **3SEB** | **24** | 0.994 | **-6** | 0.98 |

**Table S2.** Model performance indicators (RMSD, bias, NSE and r) of daytime LE, H, and Rn with TSEB and 3SEB (both forced with observed G) for the different seasons of blocks 3 and 4 in RIP720 for 2019 and 2020. The best resulting model indicator for each flux between TSEB and 3SEB are highlighted in orange.

| **Sites** | **Season** | **Flux** | **Model** | **RMSD (W/m^2^)** | **r (-)** | **Bias (W/m^2^)** | **NSE (-)** |
| --- | --- | --- | --- | --- | --- | --- | --- |
|  | **Vine Phenological stage** |  |  |  |  |  |  |
| **Blocks 3+4** | **Winter to Bud-Break (DOY ≤ 90 )** | **LE** | TSEB | 62 | 0.77 | -49 | 0.27 |
|  |  |  | **3SEB** | **47** | **0.92** | **-35** | **0.44** |
|  |  | **H** | TSEB | 44 | 0.82 | **14** | **0.65** |
|  |  |  | **3SEB** | **38** | **0.87** | 21 | **0.67** |
|  |  | **Rn** | TSEB | 36 | 0.997 | -35 | 0.93 |
|  |  |  | **3SEB** | **18** | **0.997** | **-15** | **0.98** |
|  | **Spring/bloom and berry development (DOY ~90 to ~150)** | **LE** | TSEB | 76 | 0.73 | -32 | 0.48 |
|  |  |  | **3SEB** | **57** | **0.81** | **-18** | **0.63** |
|  |  | **H** | TSEB | 68 | 0.63 | **10** | 0.33 |
|  |  |  | **3SEB** | **57** | **0.71** | 16 | **0.39** |
|  |  | **Rn** | TSEB | 22 | 0.999 | -21 | 0.98 |
|  |  |  | **3SEB** | **9** | **0.999** | **-2** | **1.00** |
|  | **Summer to Fall/Grape bunch closure through Veraison, Harvest and Senescence (DOY ~150 to ~330)** | **LE** | TSEB | 43 | **0.96** | 15 | **0.90** |
|  |  |  | **3SEB** | **40** | 0.95 | **7** | 0.89 |
|  |  | **H** | TSEB | 44 | **0.90** | -20 | 0.10 |
|  |  |  | **3SEB** | **41** | 0.86 | **1** | **0.39** |
|  |  | **Rn** | TSEB | **14** | **0.998** | **-5** | 0.99 |
|  |  |  | **3SEB** | 17 | 0.997 | 9 | 0.99 |
|  | **Fall to Winter Leaf-off (DOY ~330 to 365)** | **LE** | TSEB | 29 | 0.91 | **-22** | **0.66** |
|  |  |  | **3SEB** | **25** | **0.94** | **-17** | **0.80** |
|  |  | **H** | TSEB | 29 | 0.90 | -19 | **0.48** |
|  |  |  | **3SEB** | **21** | **0.90** | **-6** | 0.46 |
|  |  | **Rn** | TSEB | 42 | 0.999 | -41 | 0.83 |
|  |  |  | **3SEB** | **25** | 0.99/ | **-23** | **0.94** |
|  | **All seasons** | **LE** | TSEB | 53 | 0.943 | -3 | **0.88** |
|  |  |  | **3SEB** | **43** | **0.938** | **1** | 0.88 |
|  |  | **H** | TSEB | 49 | 0.76 | -10 | 0.43 |
|  |  |  | **3SEB** | **44** | **0.83** | **5** | **0.54** |
|  |  | **Rn** | TSEB | 21 | 0.997 | -13 | 0.99 |
|  |  |  | **3SEB** | **16** | 0.997 | **5** | **0.99** |
